# RAF1 Promotes Gstt1‐Mediated Ferroptosis by Promoting KDM1A Recruitment to Facilitate Pilocarpine‐Induced Acute Seizure‐Associated Neuronal Injury

**DOI:** 10.1002/cns.71177

**Published:** 2026-10-10

**Authors:** Tiantian Lu, Yonghong Feng, Xiuling Chen, Liyan Shen, Min Liu, Yanping Jia

**Affiliations:** ^1^ Department of Neonatology Haikou Maternal and Child Health Hospital Haikou China; ^2^ Department of Pediatrics The First Affiliated Hospital of Hainan University Haikou China; ^3^ Department of Pediatrics Haikou Maternal and Child Health Hospital Haikou China

**Keywords:** ferroptosis, Gstt1, KDM1A, neuronal injury, RAF1, seizures

## Abstract

**Background:**

Seizures can cause progressive neuronal damage. Our previous studies found that RAF1 mediates vitamin D's alleviation of seizures, but the mechanism by which RAF1 acts in seizures remains unclear.

**Methods:**

The pilocarpine‐induced acute seizure‐associated neuronal injury (Pilo‐ASNI) model and glutamate‐treated HT22 cells were used. Behavioral assessments were performed to evaluate seizure severity and latency; histopathological staining was performed to examine neuronal damage and survival; and cellular assays were performed to assess cell viability, cytotoxicity, and DNA damage. Transcriptome sequencing, Co‐immunoprecipitation, molecular docking, ChIP‐qPCR, and ferroptosis indicators were employed to elucidate the molecular mechanism.

**Results:**

RAF1 expression was markedly upregulated in the Pilo‐ASNI model and glutamate‐treated HT22 cells. RAF1 knockdown attenuated seizure severity and neuronal damage. Mechanistic studies in HT22 cells revealed that RAF1 inhibited Gstt1 transcription and promoted ferroptosis by promoting KDM1A recruitment and reducing H3K4me2 levels in the promoter region of Gstt1. Disrupting the RAF1‐KDM1A interaction could inhibit ferroptosis and alleviate nerve damage, whereas Gstt1 knockdown reverses the protective effect of RAF1 inhibition.

**Conclusion:**

This study revealed a RAF1‐driven pathway involving KDM1A‐mediated epigenetic suppression of Gstt1 and subsequent ferroptosis, which may represent a potential target for neuroprotective intervention in seizures.

## Graphical abstract

In the hippocampus of mice with pilocarpine‐induced acute seizures and in glutamate‐treated neuronal cell lines, the mRNA and protein levels of RAF1 were significantly increased. RAF1 interacts with the KDM1A and recruits it to the promoter of Gstt1, thereby inhibiting the transcription of Gstt1. The decreased expression of Gstt1 induces ferroptosis, thereby participating in acute seizure‐associated neuronal injury. Based on previous studies, this research further clarified the mechanism by which RAF1 contributes to acute seizure‐associated neuronal injury and provided new theoretical insights into its pathogenesis.

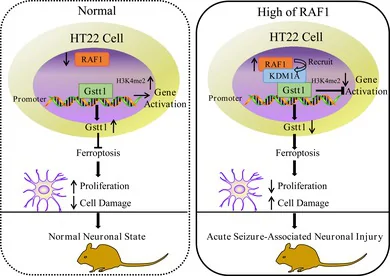

## 1 Introduction

Epilepsy is a complex chronic neurological disease. Its recurrent and spontaneous seizures seriously damage the quality of life of patients and bring a heavy disease burden to society [1, 2]. Although existing antiepileptic drugs can control the symptoms of some patients, about 30% of patients still develop refractory epilepsy, and the current treatment strategies have limited effects in preventing progressive neuronal damage related to epilepsy [3, 4, 5]. Therefore, it is of great clinical significance to reveal the molecular mechanism of seizures and secondary brain injury and to find new intervention targets.

In recent years, ferroptosis, as a type of iron‐dependent, lipid peroxidation‐driven programmed cell death [6], has been proven to play an important role in a variety of neurodegenerative diseases and nervous system injuries [7, 8]. In epilepsy models, significant changes in ferroptosis‐related markers have been observed, suggesting that it may be involved in neuronal injury after seizures [9, 10]. However, the key upstream signaling pathways and their precise mechanisms that regulate ferroptosis in seizures remain to be elucidated.

RAF1 is a core member of the mitogen‐activated protein kinase (MAPK) signaling pathway [11]. It is traditionally known for its regulatory role in cell proliferation, migration, invasion, and survival. Its abnormal function is closely related to the occurrence and development of various tumors [12, 13, 14]. Interestingly, our previous study showed that vitamin D alleviated seizure symptoms in mice by downregulating RAF1 expression and activating the mTOR signaling pathway [15], suggesting that RAF1 may exert a pro‐injury function in seizure pathology. However, the previous study only identified RAF1 as an effector target of vitamin D, without elucidating the downstream mechanisms of RAF1 itself in seizure neuronal injury. In particular, whether RAF1 regulates neuronal ferroptosis remains unknown.

Based on this, this study aims to systematically explore the role and mechanism of RAF1 in neuronal damage in seizures. Distinct from the previous work focusing on vitamin D targets, this study, through transcriptome sequencing, in vivo and in vitro functional assays, and epigenetic analysis, revealed a complete signaling axis in which RAF1 suppresses Gstt1 transcription by promoting the recruitment of KDM1A, thereby driving neuronal ferroptosis, providing new experimental evidence and drug targets for the treatment of seizures.

## 2 Materials and Methods

### 2.1 Study Design

This study investigated the role of RAF1 in epileptic neuronal injury and its underlying mechanisms using a pilocarpine‐induced acute seizure‐associated neuronal injury (Pilo‐ASNI) model and glutamate‐treated HT22 cells. In vivo, RAF1 or Gstt1 knockdown was assessed for seizure severity, neuronal damage, and ferroptosis. In vitro, HT22 cells with RAF1/Gstt1 knockdown or K277A mutant were evaluated for cellular phenotypes, with epigenetic mechanisms primarily dissected in cells. All animal handling and experimental protocols were reviewed and approved by the Animal Use and Care Committee of Hainan University. Primers for quantitative real‐time PCR (qRT‐PCR) are shown in Table S1. Other detailed informations are available in the Supporting Information.

### 2.2 Statistical Analysis

All data are expressed as mean ± standard deviation and are derived from at least three independent experiments. All outcome assessments, including histology, behavioral tests, and biochemical assays, were performed by investigators blinded to the group allocation. Statistical analysis was performed using GraphPad Prism 8.0. Student's *t*‐test was used for comparison between two groups, and one‐way ANOVA and Tukey's post hoc test were used for comparison among multiple groups. A *p*‐value of < 0.05 was considered statistically significant.

## 3 Results

### 3.1 RAF1 Knockdown Alleviates Epileptic Seizures and Neuronal Damage in Pilo‐ASNI Mice

To explore the role of RAF1 in seizures, we first established a Pilo‐ASNI mouse model and knocked down RAF1 in the hippocampus via LV‐shRNA (Figure 1A). Behavioral analysis showed that, compared with the LV‐sh‐NC group, RAF1 knockdown markedly prolonged the latency to convulsions during the acute phase of the model (Figure 1B) and attenuated the seizure severity during the subsequent observation period (Figure 1C). Additionally, we confirmed that RAF1 was elevated in the hippocampus of model mice, while LV‐sh‐RAF1 treatment effectively inhibited its expression level (Figure 1D,E).

**FIGURE 1:**
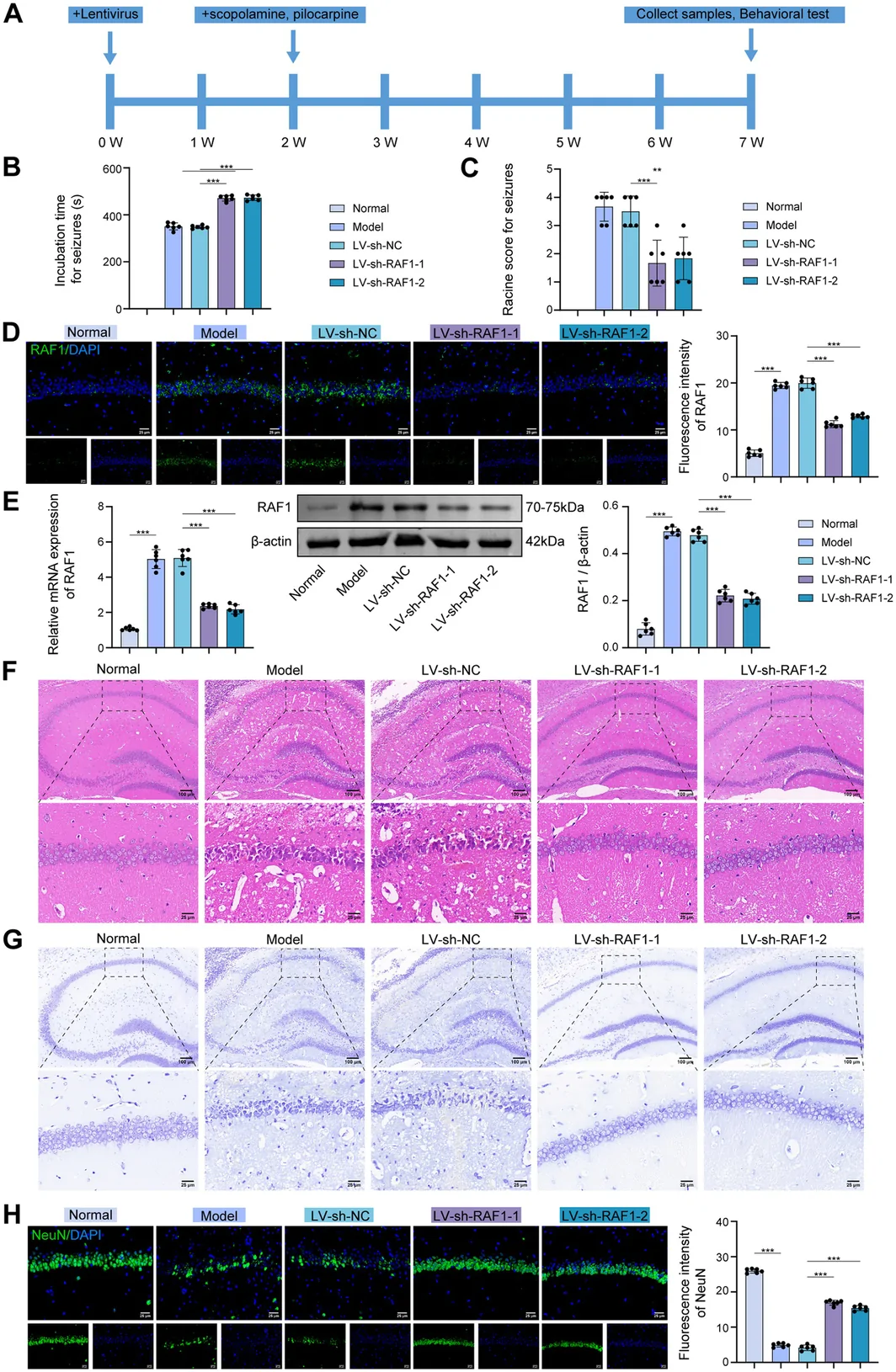
RAF1 knockdown alleviates seizures and neuronal damage in Pilo‐ASNI mice. (A) Animal handling experimental protocol. (B) Latency of seizure onset. (C) Seizure severity was scored according to the Racine scale. (D) IF detection of RAF1 expression in CA1 region of hippocampus. Scale bar = 25 μm. (E) qRT‐PCR and WB were used to detect the expression of Raf1. (F) Representative images of H&E staining in CA1 region of hippocampus. (G) Representative images of Nissl staining in CA1 region of hippocampus. (H) Representative immunofluorescence images and statistical analysis of NeuN in CA1 region of hippocampus. Scale bar = 25 μm. *n* = 6. **p < 0.01; ***p < 0.001.

Next, we evaluated the effect of RAF1 knockdown on hippocampal neuronal damage. H&E staining identified that neurons in the CA1 region of model mice were disordered, reduced in number, and exhibited obvious nuclear pyknosis, while RAF1 knockdown markedly improved neuronal morphology and reduced cell loss (Figure 1F). Nissl staining further confirmed that RAF1 knockdown increased the number of surviving neurons in the CA1 region (Figure 1G). Moreover, IF staining identified that NeuN‐positive cells were significantly increased in the RAF1 knockdown group compared with the LV‐sh‐NC group (Figure 1H). Collectively, these results suggest RAF1 downregulation reduces pilocarpine‐induced seizure severity and alleviates hippocampal neuronal damage.

### 3.2 Downregulation of RAF1 Ameliorates Glutamate‐Induced HT22 Cell Injury

To evaluate the effect of RAF1 in vitro, we first examined its colocalization with different cell‐type markers in the mouse hippocampus by IF staining. RAF1 predominantly colocalized with the neuronal marker NeuN, showed weaker colocalization with the astrocytic marker GFAP and inhibitory neuronal marker GAD67, and little to none with the microglial marker Iba1 (Figure S1), suggesting that RAF1 is primarily expressed in excitatory neurons. Accordingly, we selected the mouse hippocampal neuronal cell line HT22 as an in vitro model and screened shRNA constructs targeting RAF1 in these cells. All three shRNA sequences effectively reduced RAF1 mRNA and protein levels in HT22 cells; sh‐RAF1#2, with the highest efficiency, was used for subsequent experiments (Figure 2A). Glutamate treatment markedly elevated RAF1 expression (Figure 2B). RAF1 knockdown markedly enhanced cell viability, reduced LDH release (Figure 2C,D), inhibited cell death (Figure 2E), and suppressed 8‐OHdG production (Figure 2F), indicating alleviation of glutamate‐induced neuronal injury and DNA oxidative damage.

**FIGURE 2:**
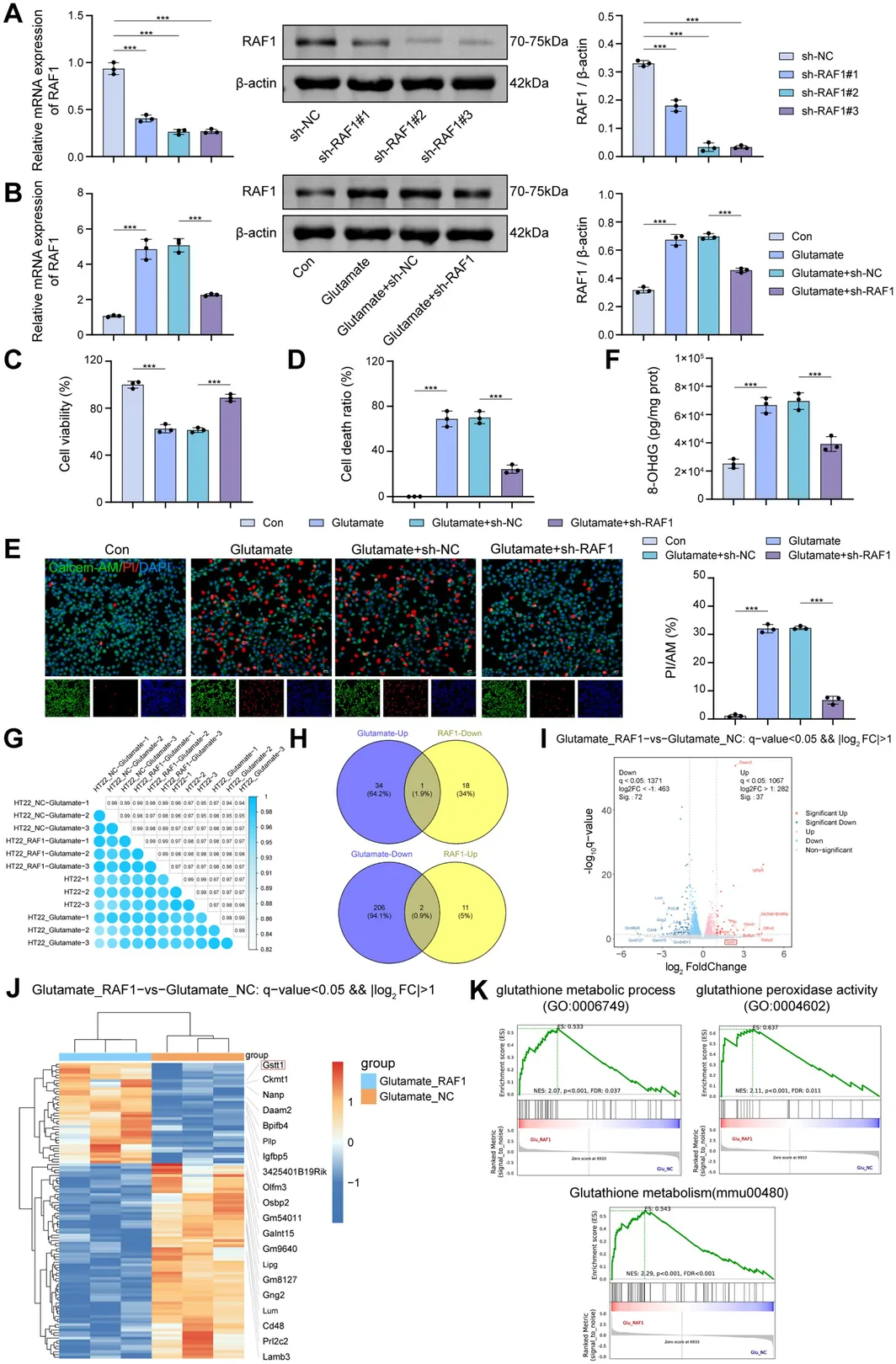
Downregulation of RAF1 ameliorates glutamate‐induced HT22 cell injury and identifies Gstt1 as a key transcriptional target. (A and B) qRT‐PCR and WB were used to detect the expression of RAF1. (C) CCK‐8 assay. (D) LDH cytotoxicity assay. (E) Representative images and statistical analysis of Calcein‐AM/PI staining. Scale bar = 50 μm. (F) 8‐OHdG level measurement of DNA damage in cells. (G) Sample correlation heatmap. (H) Venn Diagram. (I) Volcano plot of differentially expressed genes. (J) Heatmap of differentially expressed genes. (K) GSEA Analysis. *n* = 3. ***p < 0.001.

To validate the above findings under pathophysiological conditions more closely resembling epileptiform discharges, we treated primary hippocampal neurons with low‐Mg^2+^ extracellular solution ([Mg^2+^]_0_), as described by Miranda Mele et al. [16], to model excitotoxic injury induced by seizure‐like activity. qRT‐PCR results showed that RAF1 mRNA levels were significantly elevated in the [Mg^2+^]_0_‐treated primary neurons and reversed by RAF1 knockdown (Figure S2A). RAF1 knockdown also attenuated [Mg^2+^]_0_‐induced calcium overload (Figure S2B), increased cell viability, reduced LDH release (Figure S2C,D), and alleviated neuronal death and oxidative DNA damage in primary neurons (Figure S2E,F). Collectively, these results demonstrate that RAF1 downregulation effectively attenuates excitotoxic neuronal injury in both glutamate‐treated HT22 cells and low‐Mg^2+^‐treated primary hippocampal neurons.

### 3.3 RNA‐Seq Analysis of the Intervention Mechanism of RAF1 Downregulation on Glutamate‐Induced Neuronal Damage

To examine the molecular mechanism of RAF1 regulating neuronal injury in seizures, HT22 cells with glutamate and sh‐RAF1 were subjected to RNA‐seq (Figure 2G–K). Glutamate induced extensive transcriptional changes, which were reversed by RAF1 knockdown. Venn diagram analysis identified Prl2c2 as upregulated by glutamate and downregulated by RAF1 knockdown, and Ckmt1 and Gstt1 as downregulated by glutamate and restored by RAF1 knockdown (Figure 2H). Volcano plot and heatmap further identified that the expression of Gstt1 was effectively restored after RAF1 knockdown (Figure 2I,J). GSEA showed that RAF1 knockdown markedly enriched “glutathione metabolic process” and “glutathione peroxidase activity” pathways (Figure 2K). Considering that the core features of ferroptosis are glutathione depletion and GPX4 inactivation [17], and that Gstt1, as a member of the glutathione S‐transferase family, has the primary function of protecting cells from oxidative damage by catalyzing the binding of glutathione to harmful substances [18], studies have shown that Gstt1 deficiency can weaken cellular antioxidant capacity [19], and its expression is associated with the occurrence of ferroptosis [20]. Therefore, we chose Gstt1 as a key target for subsequent research on the regulation of neuronal antioxidant capacity and ferroptosis downstream of RAF1.

### 3.4 RAF1 Participates in HT22 Cell Injury by Promoting Ferroptosis Through Inhibiting Gstt1 Expression

Based on the RNA‐seq results, we verified the regulation of Gstt1 by RAF1. In HT22 cells, glutamate treatment markedly inhibited Gstt1 expression, while RAF1 knockdown effectively restored its mRNA and protein levels (Figure 3A), consistent with in vivo findings (Figure 3B). To determine whether Gstt1 is sufficient to rescue glutamate‐induced neuronal injury, we overexpressed Gstt1 in HT22 cells (Figure S3A). The results showed that Gstt1 overexpression significantly increased cell viability and reduced LDH release (Figure S3B,C), confirming its protective role.

**FIGURE 3:**
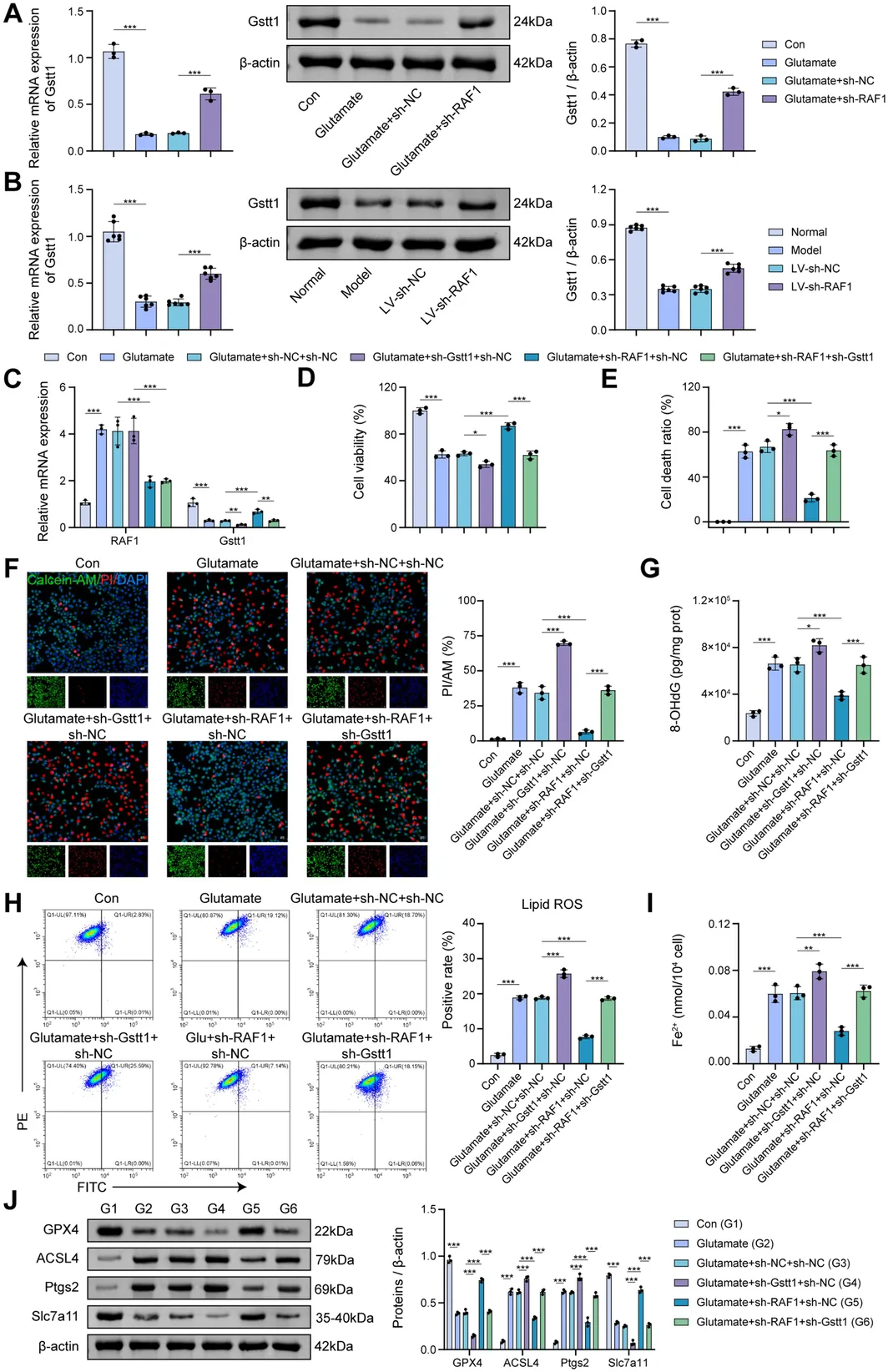
RAF1 participates in HT22 cell injury by promoting ferroptosis through inhibiting Gstt1 expression. (A) The expression of Gstt1 in each group of cells was detected by qRT‐PCR and WB. (B) Expression of Gstt1 in animal tissues was detected by qRT‐PCR and WB. (C) qRT‐PCR detection of *RAF1* and *Gstt1* expression levels. (D) CCK‐8 assay. (E) LDH cytotoxicity assay. (F) Representative images and statistical analysis of Calcein‐AM/PI staining. Scale bar = 50 μm. (G) 8‐OHdG level measurement of DNA damage in cells. (H) Representative images and statistical analysis of intracellular lipid ROS levels assessed by flow cytometry. (I) The kit was used to detect the content of Fe^2+^ in cells. (J) The expression of ferroptosis‐related proteins was detected by WB. *n* = 3. *p < 0.05; **p < 0.01; ***p < 0.001.

Co‐knockdown experiments showed that RAF1 knockdown effectively reversed glutamate‐induced expression changes in RAF1 and Gstt1, whereas Gstt1 co‐knockdown specifically blocked Gstt1 recovery without affecting RAF1 (Figure 3C). The protective effect of RAF1 knockdown on viability, LDH release, cell death, and oxidative DNA damage was significantly reversed by Gstt1 co‐knockdown (Figure 3D–G).

Regarding ferroptosis, Gstt1 overexpression significantly attenuated glutamate‐induced Fe^2+^ accumulation, restored GPX4 and Slc7a11, downregulated ACSL4 and Ptgs2 (Figure S3D,E), and elevated the GSH/GSSG ratio and GPX activity (Figure S3F,G), indicating that restoration of Gstt1 expression effectively counteracts glutamate‐induced neuronal injury and ferroptosis. To further validate these findings, we examined the effect of Gstt1 knockdown in the context of RAF1 knockdown. RAF1 knockdown similarly inhibited lipid ROS and Fe^2+^, restored ferroptosis‐related proteins, and improved glutathione metrics, effects that were significantly reversed by Gstt1 co‐knockdown (Figures 3H–J and S3H,I). These data indicate that RAF1 promotes ferroptosis via Gstt1 suppression, exacerbating glutamate‐induced neuronal injury.

### 3.5 RAF1 Suppresses Gstt1 Expression by Promoting KDM1A Recruitment to Block H3K4me2 Modification

RAF1 was predominantly cytoplasmic in resting HT22 cells but accumulated in the nucleus upon glutamate stimulation (Figure 4A). Given that RAF1 knockdown increased Gstt1 mRNA, we hypothesized that nuclear RAF1 transcriptionally regulates Gstt1. UCSC database analysis revealed a marked H3K4me2/3 peak in the Gstt1 promoter (Figure 4B), and BioGRID database prediction together with Co‐IP experiments confirmed a specific interaction between RAF1 and the demethylase KDM1A (Figure 4C,D).

**FIGURE 4:**
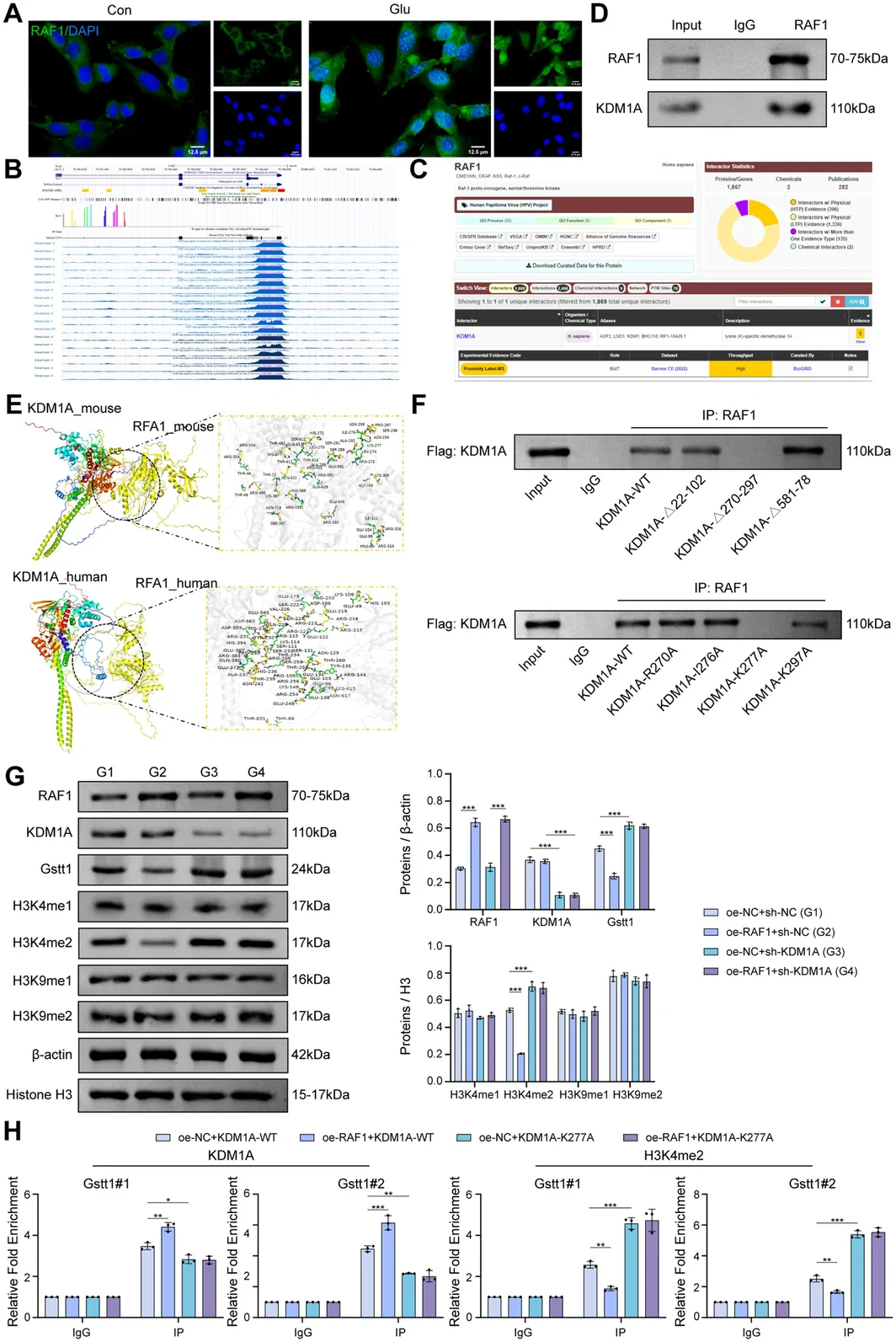
RAF1 suppresses Gstt1 expression by promoting KDM1A recruitment to block H3K4me2 modification. (A) IF Analysis of the Subcellular Localization of RAF1 in Cells. Scale bar = 12.5 μm. (B) UCSC database shows peak. (C) The BioGRID database predicted potential histone demethylases that bind to RAF1. (D) CO‐IP assay. (E) Molecular docking analysis. (F) CO‐IP assay. (G) WB detection of the levels of RAF1, KDM1A, Gstt1, H3K4me1/2 and H3K9me1/2. (H) CHIP‐qPCR was used to detect the enrichment changes of KDM1A and H3K4me2 antibodies in the promoter region of Gstt1. *n* = 3. *p < 0.05; **p < 0.01; ***p < 0.001.

To further elucidate the molecular basis of this interaction, we performed docking simulations between RAF1 and KDM1A proteins using the ClusPro server. The top‐ranked docking model achieved a Docking Score of −1250.5 kcal/mol, indicating a strong binding affinity between the two proteins. This low docking score provides structural prediction evidence supporting the notion that RAF1 may recruit KDM1A to negatively regulate Gstt1 expression. As shown in the figure, the protein KDM1A interacts with the protein RAF1 through hydrogen bonds and hydrophobic interactions. The detailed residue pairs are listed in the Table S2. In addition, the Docking Score of the human homologous protein docking is −1424.3 kcal/mol, and there are also multiple interaction sites, suggesting a strong binding affinity between RAF1 and KDM1A (Figure 4E). Truncation and point mutation analyses identified that the K277A mutation in KDM1A disrupted its interaction with RAF1 (Figure 4F).

Functionally, RAF1 overexpression in HT22 cells reduced Gstt1 protein level and H3K4me2 modification, which were reversed by KDM1A knockdown without affecting H3K4me1 or H3K9me1/2 (Figure 4G). ChIP‐qPCR experiments confirmed that RAF1 overexpression facilitated KDM1A enrichment at the Gstt1 promoter and reduced H3K4me2 levels; notably, when KDM1A‐RAF1 interaction was disrupted by the K277A mutation, RAF1 re‐overexpression failed to promote KDM1A enrichment or reduce H3K4me2 levels at the Gstt1 promoter (Figure 4H), indicating that the function of RAF1 requires KDM1A recruitment. Collectively, these findings suggest that RAF1 interacts with KDM1A and facilitates its recruitment to the Gstt1 promoter, thereby inhibiting Gstt1 transcriptional expression via reduced H3K4me2 modification.

### 3.6 RAF1 Participates in HT22 Cell Injury by Recruiting KDM1A to Promote Ferroptosis Through Inhibiting Gstt1 Expression

To probe whether RAF1 function depends on KDM1A recruitment, we first confirmed that KDM1A wild‐type (WT) and K277A mutant exhibited comparable expression (Figure S4A), predominant nuclear localization, and similar protein stability (Figure S4B,C). In glutamate‐treated HT22 cells, RAF1 overexpression alone markedly aggravated glutamate‐induced cell damage, as evidenced by attenuated cell viability, augmented LDH release, elevated cell death and DNA oxidative damage (Figure 5A–D), along with deteriorated ferroptosis indicators (Figures 5E–G and S4D,E). In contrast, the KDM1A‐K277A mutant alone exerted a protective effect, effectively reversing these injury indicators. Notably, when RAF1 was overexpressed in the presence of the KDM1A‐K277A mutant, RAF1 overexpression no longer induced ferroptosis or cell damage (Figures 5A–G and S4D,E). These phenomena indicate that the pro‐ferroptotic and neurotoxic effects of RAF1 in excitotoxic injury are strictly dependent on KDM1A recruitment, identifying KDM1A as a key downstream executor of RAF1 function.

**FIGURE 5:**
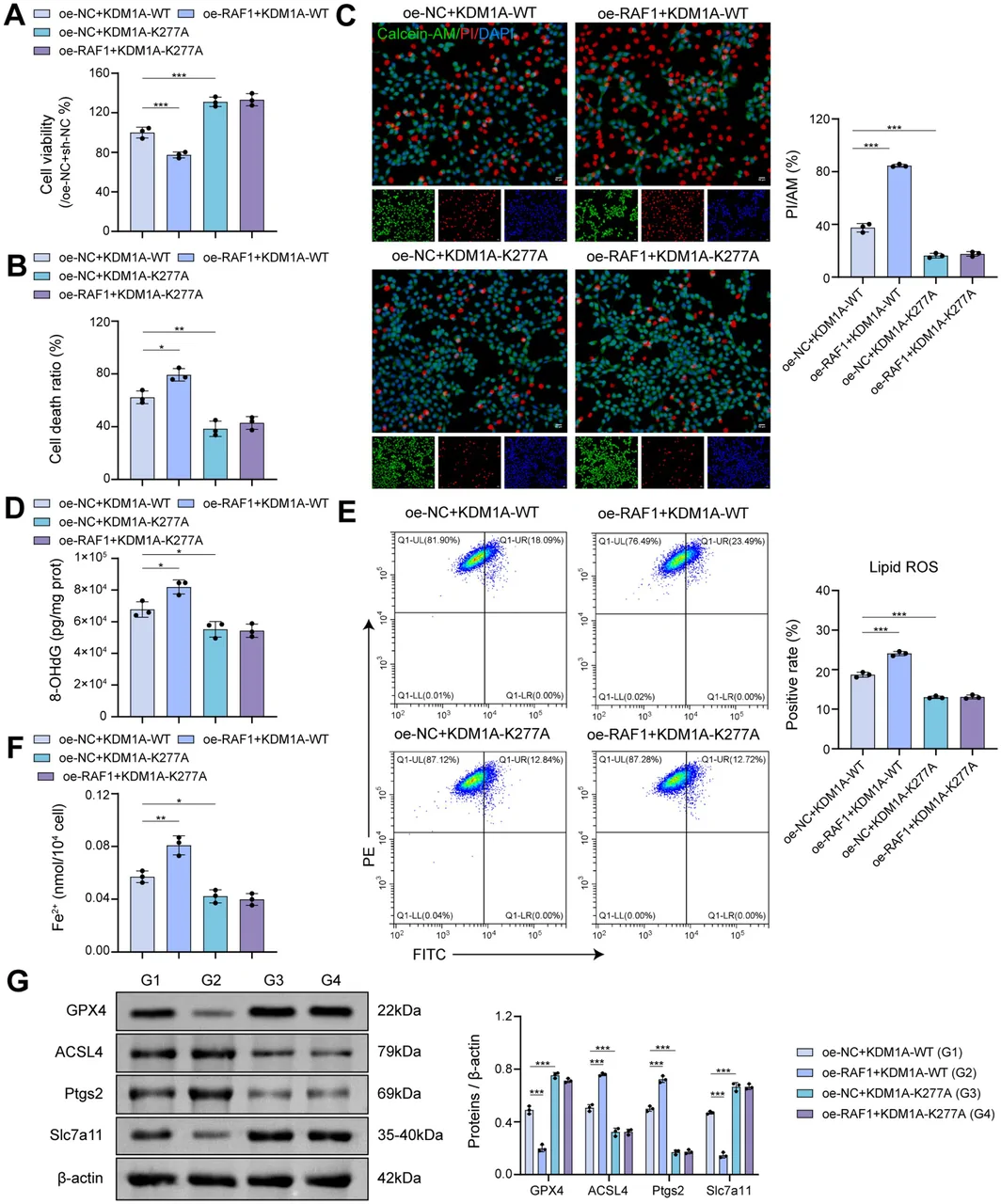
RAF1 participates in HT22 cell injury by recruiting KDM1A to promote ferroptosis though inhibiting Gstt1 expression. (A) CCK‐8 assy. (B) LDH cytotoxicity assay. (C) Representative images and statistical analysis of Calcein‐AM/PI staining. Scale bar = 50 μm. (D) 8‐OHdG level measurement of DNA damage in cells. (E) Representative images and statistical analysis of intracellular lipid ROS levels assessed by flow cytometry. (F) The kit was used to detect the content of Fe^2+^ in cells. (G) The expression of ferroptosis‐related proteins were detected by WB. *n* = 3. *p < 0.05; **p < 0.01; ***p < 0.001.

### 3.7 RAF1 Exacerbates Pilocarpine‐Induced Acute Seizure‐Associated Neuronal Injury by Inducing Gstt1‐Mediated Ferroptosis

To assess how RAF1 and Gstt1 cooperate functionally in vivo, we injected lentivirus into the hippocampus to knock down Gstt1 (LV‐sh‐Gstt1), knock down RAF1 (LV‐sh‐RAF1), or knock down both (LV‐sh‐RAF1 + LV‐sh‐Gstt1) in Pilo‐ASNI model mice. Gstt1 knockdown shortened the latency to convulsions during the acute phase of the model and exacerbated the seizure severity during the subsequent observation period, while RAF1 knockdown exerted a protective effect that was significantly reversed by Gstt1 co‐knockdown (Figure 6A,B). Molecular detection confirmed Raf1 and Gstt1 expression in the hippocampus of each group of animals (Figure 6C).

**FIGURE 6:**
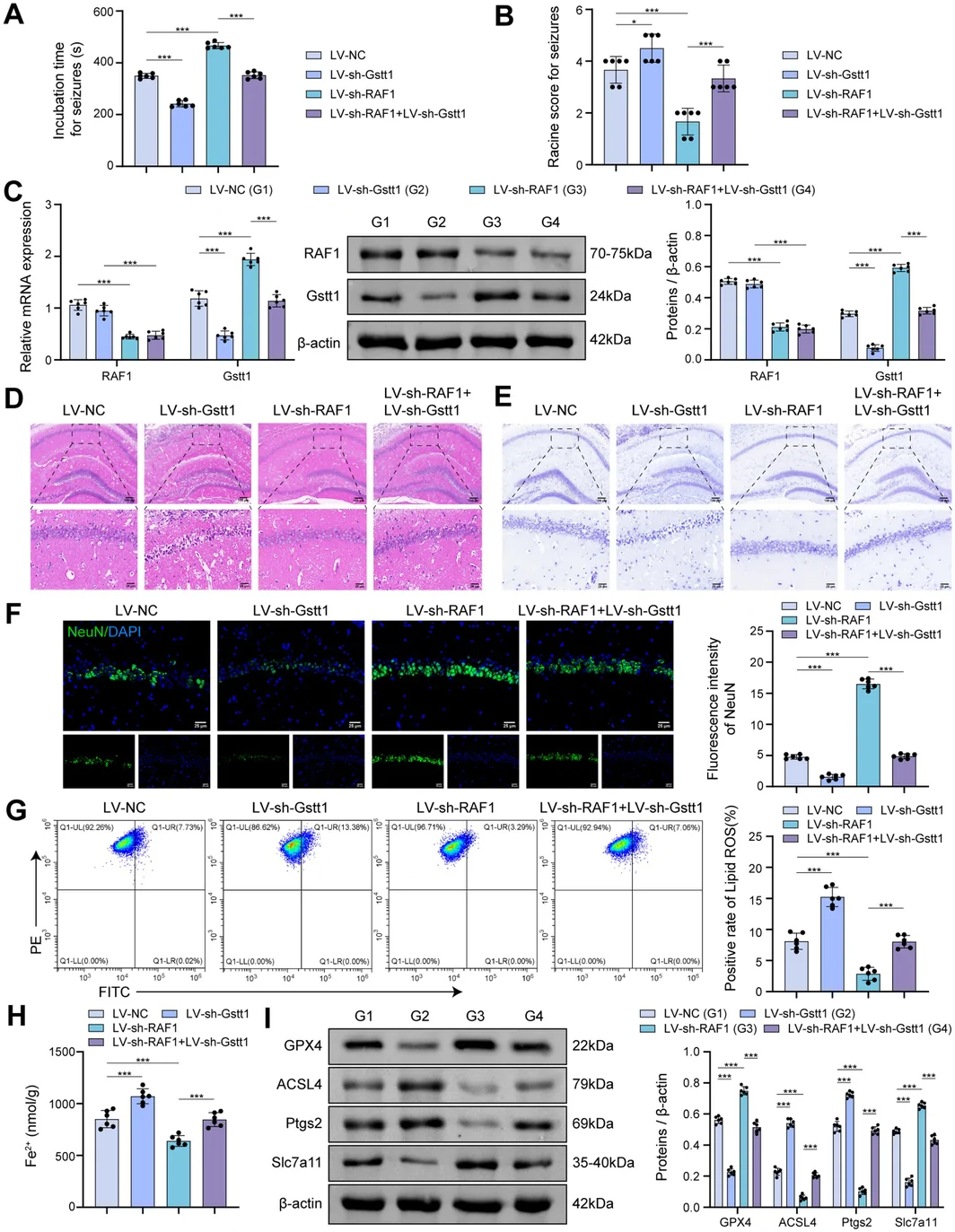
RAF1 exacerbates pilocarpine‐induced acute seizure‐associated neuronal injury by inducing Gstt1‐mediated ferroptosis. (A) Latency of seizures onset. (B) Seizures severity was scored according to the Racine scale. (C) qRT‐PCR and WB were used to detect the expression of Raf1 and Gstt1. (D) Representative images of H&E staining in CA1 region of hippocampus. Upper Scale bar = 100 μm; Lower Scale bar = 25 μm. (E) Representative images of Nissl staining in CA1 region of hippocampus. (F) Representative immunofluorescence images and statistical analysis of NeuN in CA1 region of hippocampus. Scale bar = 25 μm. (G) Representative images and statistical analysis of intracellular lipid ROS levels assessed by flow cytometry. (H) The kit was used to detect the content of Fe^2+^ in cells. (I) The expression of ferroptosis‐related proteins were detected by WB. *n* = 6. *p < 0.05; ***p < 0.001.

Histologically, Gstt1 knockdown exacerbated CA1 neuronal loss, while RAF1 knockdown markedly alleviated it, and this protective effect was significantly reversed by Gstt1 co‐knockdown (Figure 6D–F). In addition, RAF1 knockdown effectively reduced lipid ROS and Fe^2+^, and elevated GSH/GSSG ratio and GPX activity, whereas Gstt1 knockdown showed opposite effects; co‐knockdown almost completely reversed the anti‐ferroptotic effects of RAF1 knockdown (Figures 6G–I and S4F,G). These phenomena demonstrate that RAF1 mainly regulates ferroptosis by regulating Gstt1 expression in vivo, modulating acute seizure‐associated neuronal injury.

## 4 Discussion

Epilepsy is a chronic neurological disease characterized by abnormal excessive synchronous discharge of neurons [21, 22]. Its pathogenesis is complex, but in recent years, effective treatment methods for neuronal damage caused by epileptic seizures are still scarce [23]. By integrating transcriptome sequencing and in vivo (Pilo‐ASNI model) and in vitro (glutamate‐treated HT22 cells) models, this study reveals a novel mechanism by which RAF1 epigenetically suppresses Gstt1 expression through facilitating KDM1A recruitment, a process mechanistically defined in HT22 cells that may drive ferroptosis and contribute to seizure‐associated neuronal injury. These findings provide a new theoretical framework for understanding the pathology of seizures.

Previous studies have shown that RAF1 dysfunction is associated with a variety of diseases. In oncology, RAF1 fusions [24] and kinase activity [25] are therapeutic targets; in RASopathies, its mutations cause cardiac and growth abnormalities [26, 27]; and in neurodegeneration, RAF1 acts via MAPK, VEGF and other pathways [28]. Here, RAF1 was upregulated in seizure models and glutamate‐treated neurons, and its knockdown attenuated seizure severity, prolonged the latency period, and markedly improved neuron survival, revealing a pro‐injury role of RAF1 in seizures. Additionally, glutamate‐induced RAF1 nuclear translocation suggests non‐canonical nuclear functions beyond its cytoplasmic kinase activity, further expanding the understanding of RAF1's functional diversity and disease specificity.

Glutathione S‐transferases (GSTs) are key enzymes in glutathione (GSH) metabolism, with Gstt1 belonging to the drug‐metabolizing enzyme II family [29]. Here, transcriptome sequencing revealed Gstt1 as a key gene regulated by RAF1 in glutamate‐induced excitotoxicity. RAF1 knockdown significantly reversed the glutamate‐stimulated Gstt1 suppression. GSEA showed enrichment of glutathione metabolic pathways, closely related to the core characteristics of ferroptosis, GSH depletion [30] and GPX4 inactivation [31]. Given Gstt1's role in glutathione conjugation [18, 19], we selected it as a downstream target of RAF1‐mediated neuronal ferroptosis. This finding echoes the known function of GST family enzymes in regulating ferroptosis. GST loss impairs ROS clearance, disrupts redox balance, and drives lipid peroxidation [19, 32], potentially inducing ferroptosis. For example, Gstt1/Gstm1 deficiency can aggravate ferroptosis in cisplatin‐induced acute kidney injury [20]; in the heat stress model, the decrease of Gstt1 expression in broiler muscle tissue is accompanied by the activation of ferroptosis, while its expression is restored and ferroptosis is reduced after selenium nanoparticle intervention [33]. These results indicate that Gstt1 has a cross‐tissue conserved function in maintaining redox homeostasis and resisting ferroptosis. In addition, further functional experiments confirmed that knocking down Gstt1 could reverse the neuroprotective and ferroptosis‐inhibitory effects brought by RAF1 knockdown, establishing the core position of Gstt1 in this pathway. Notably, Gstt1 knockdown almost completely reversed the neuroprotective effects of RAF1 knockdown, restoring the injury levels to those comparable to the control group. This indicates that, in this context, Gstt1 serves as the primary downstream effector of RAF1‐mediated protection. Although minor contributions from Gstt1‐independent mechanisms cannot be entirely excluded, the nearly complete reversal demonstrates that RAF1 functions predominantly through Gstt1, positioning this axis as a linear signaling cascade rather than parallel pathways, and further establishing Gstt1 as a key nodal point of this pathway. Collectively, our results demonstrate that RAF1 knockdown protects neurons from excitotoxic injury through the regulation of Gstt1, accompanied by modulation of multiple ferroptosis‐associated features. Beyond GST‐mediated glutathione metabolism, broader antioxidant defense systems may also contribute to the regulation of ferroptosis susceptibility in epilepsy models. Notably, Nrf2‐dependent redox responses [34, 35, 36] and thioredoxin‐based neuroprotective pathways [37] have been implicated in counteracting oxidative injury and may intersect with Gstt1‐regulated glutathione homeostasis to collectively modulate ferroptosis in neurons. Some studies have also shown that certain GST subtypes (such as Gstp1) may form a GPX4‐independent defense mechanism through synergy with transporters (RLIP76/MRP1) [38] or by regulating signaling pathways such as JNK/c‐Jun [39]. Therefore, future studies can further explore the main way in which Gstt1 exerts its anti‐ferroptosis effect, which will help to more fully understand its protective mechanism in seizure‐related neuronal damage.

Histone methylation is an important epigenetic mechanism in epilepsy [40], dynamically regulating neuronal plasticity and excitability [41]. KDM1A, the first identified histone lysine‐specific demethylase [42], removes H3K4me1/me2 to suppress gene expression [43, 44, 45]. Interestingly, our cellular‐level studies found that the regulatory effect of RAF1 on Gstt1 depends on KDM1A. Mechanistically, RAF1 promotes KDM1A enrichment at the Gstt1 promoter, reduces H3K4me2 levels, and suppresses Gstt1 transcription, with K277 identified as the key site for KDM1A‐RAF1 interaction. This is consistent with the known KDM1A mechanism. For example, in breast cancer, on the one hand, HER2 upregulation can activate KDM1A by affecting ATF7, changing the modification levels of H3K9me2 and H3K4me2 [46]; on the other hand, the transcriptional repressor SNAI2 directly recruits KDM1A to the ERα promoter region, and inhibits ERα expression by removing the H3K4me2 mark [47]. This indicates that KDM1A may be a common molecular hub connecting diverse pathological signals and epigenetic silencing. Functionally, transfecting the KDM1A‐K277A mutant plasmid alleviated glutamate‐induced ROS accumulation, neuronal DNA damage, and ferroptosis. This result is closely related to the complex role of KDM1A in regulating oxidative stress and cell death. In some pathological models, inhibition of KDM1A may also play a protective role. For example, KDM1A has been shown to exert antioxidant and pro‐survival effects in tumors by epigenetically regulating metabolic pathways to reduce ROS levels and lipid peroxidation or by promoting SLC7A11 SUMOylation to inhibit ferroptosis [48]. These findings imply that KDM1A mainly plays an antioxidant and pro‐survival role in tumor cells. This functional difference highlights the significant context‐dependent nature of the biological effects of KDM1A, suggesting that the specificity of cell types and pathological backgrounds should be fully considered when developing KDM1A‐based precision treatment strategies in the future. On top of that, we also confirmed that RAF1 could no longer inhibit Gstt1, promote injury, and induce ferroptosis when RAF1 was overexpressed in HT22 cells transfected with the KDM1A‐K277A mutant plasmid. This indicates that, at the cellular level, KDM1A is an essential mediator for RAF1 to exert its pathological function.

In vivo experiments also provided strong evidence for this pathway. Simultaneous knockdown of Gstt1 in Pilo‐ASNI model mice could partially reverse all the beneficial effects brought about by RAF1 knockdown, including behavioral improvement, neuronal protection, and ferroptosis inhibition. This result strongly proves that the pro‐epileptic effect of RAF1 in the complex biological in vivo environment strictly depends on the ferroptosis pathway mediated by Gstt1. This provides multiple potential precise targets for future therapeutic intervention: RAF1 itself, the interaction interface between RAF1 and KDM1A, the enzymatic activity site of KDM1A, and the restoration or enhancement of the function of Gstt1.

Sex differences, as a critical biological variable, have received increasing attention for their impact on various aspects of epilepsy, ranging from seizure susceptibility and neuroinflammatory responses to epileptogenesis and cognitive outcomes [49]. Clinical and preclinical evidence suggests that males generally exhibit higher seizure susceptibility [50, 51] and more pronounced neuroinflammatory responses [52], whereas females often perform better in certain cognitive domains [53] but may face different affective disorders and comorbidities [54, 55]. The underlying mechanisms are multifactorial, involving sex hormones, sex chromosome‐linked genetic factors, and their interactions with neural circuits [49]. With regard to oxidative stress, NOX2 has been identified as a major source of ROS during epilepsy [56], and its inhibition has shown therapeutic potential in modifying the epileptic course [57]. Notably, recent studies have demonstrated that sex‐dependent redox mechanisms may differentially regulate these processes, with males responding more robustly to NOX2 inhibition than females [58, 59]. In view of this evidence, the present study used only male mice, and our results may not fully reflect the sex‐dependent pathophysiological mechanisms in epilepsy. Future studies should include both sexes to determine whether the observed protective effects are sex‐independent or more beneficial in males.

This study still has the following limitations. First, the study mainly focused on the hippocampus, a key lesion area of seizures. In the future, the universality of this pathway needs to be verified in other seizure‐related brain regions such as the cortex and amygdala. Second, electroencephalographic (EEG) monitoring was not performed during animal modeling to confirm epileptiform discharges. Although behavioral assessments using the Racine scale and typical seizure characteristics strongly support the model's validity, EEG recording remains the gold standard for confirming epileptic seizures, and future studies should incorporate electrophysiological recordings to further validate our findings. Third, it should be noted that the mechanistic evidence for KDM1A recruitment and H3K4me2 changes at the Gstt1 promoter was obtained primarily from cultured cells. Although our in vivo data confirmed the regulatory relationship between RAF1 and Gstt1 in hippocampal tissues, direct in vivo intervention targeting KDM1A has not been performed in this study. Therefore, the RAF1‐KDM1A‐Gstt1 epigenetic axis currently relies mainly on in vitro evidence, and future studies using in vivo KDM1A manipulation or ChIP‐qPCR in brain tissues are warranted to further validate this mechanism. Fourthly, the precise mechanism by which RAF1 facilitates KDM1A recruitment to the Gstt1 promoter remains to be fully defined, and whether this occurs through direct binding or via intermediary factors requires further investigation using orthogonal approaches such as GST pull‐down or Isothermal Titration Calorimetry (ITC). Therefore, future studies can further explore the main way in which Gstt1 exerts its anti‐ferroptosis effect, which will help to more fully understand its protective mechanism in seizure‐related neuronal damage. Finally, to promote the clinical transformation of this discovery, it is necessary to verify the expression and changes of the RAF1‐KDM1A‐Gstt1 axis in clinical seizure patients, and explore its correlation with the severity and prognosis of the disease.

## 5 Conclusion

Our findings identify RAF1 as a key contributor to seizure‐associated neuronal injury and suggest the involvement of the KDM1A/Gstt1‐mediated ferroptotic pathway in this process. Specifically, RAF1 upregulation facilitates KDM1A recruitment to the Gstt1 promoter region, where it was found to reduce H3K4me2 levels to suppress Gstt1 transcription, which may promote ferroptosis and contribute to seizure‐associated neuronal damage. These observations provide a new perspective for understanding the molecular pathological mechanism of seizures and suggest that the RAF1‐KDM1A‐Gstt1 axis may represent a potential intervention target for further investigation in seizure therapy.

## Author Contributions

Data curation: Tiantian Lu, Yonghong Feng, Liyan Shen; Formal analysis: Tiantian Lu, Yonghong Feng, Liyan Shen; Funding acquisition: Tiantian Lu; Investigation: Yonghong Feng, Xiuling Chen; Methodology: Yanping Jia; Project administration: Yanping Jia; Supervision: Yanping Jia; Validation: Xiuling Chen, Min Liu; Visualization: Tiantian Lu, Xiuling Chen, Min Liu; Writing – original draft: Tiantian Lu; Writing – review and editing: Yanping Jia. All authors reviewed the results and approved the final version of the manuscript.

## Funding

This study was supported by Fostering Talents Project of Haikou Maternal and Child Health Hospital (No. RC24L01).

## Ethics Statement

The study was approved by the Animal Use and Care Committee of Hainan University (No. HNUAUCC‐2025‐00144). The study was performed in accordance with the ARRIVE guidelines.

## Consent

The authors have nothing to report.

## Conflicts of Interest

The authors declare no conflicts of interest.

## Supporting information


**Figure S1:** Cellular localization of RAF1 in the hippocampus of epileptic mice. (A) RAF1 with the neuronal marker NeuN. Scale bar = 25 μm. (B) RAF1 with the astrocytic marker GFAP. Scale bar = 25 μm. (C) RAF1 with the microglial marker Iba1. Scale bar = 25 μm. (D) RAF1 with the inhibitory neuronal marker GAD67. Scale bar = 25 μm. *n* = 3.
**Figure S2:** Downregulation of RAF1 ameliorates excitotoxic injury in low‐Mg^2+^‐treated primary hippocampal neurons. (A) qRT‐PCR analysis of RAF1 mRNA expression. (B) Intracellular calcium overload assessed by the Fluo‐4 AM calcium fluorescence probe. (C) CCK‐8 assay. (D) LDH cytotoxicity assay. (E) Representative images and statistical analysis of Calcein‐AM/PI staining. Scale bar = 50 μm. (F) 8‐OHdG level measurement of DNA damage in cells. *n* = 3. *p < 0.05; **p < 0.01; ***p < 0.001.
**Figure S3:** Gstt1 mediates the anti‐ferroptotic effect of RAF1 knockdown in glutamate‐treated HT22 cells. (A) qRT‐PCR and WB analysis of Gstt1 expression. (B) CCK‐8 assay. (C) LDH cytotoxicity assay. (D) The kit was used to detect the content of Fe^2+^ in cells. (E) The expression of ferroptosis‐related proteins was detected by WB. (F, H) Cellular GSH/GSSG ratio. (G, I) Cellular GPX activity. *n* = 3. *p < 0.05; **p < 0.01; ***p < 0.001.
**Figure S4:** KDM1A mediates RAF1‐mediated epigenetic regulation of Gstt1 and functional validation. (A) WB analysis of KDM1A protein expression. (B) IF staining showing the subcellular localization of KDM1A. Scale bar = μm. (C) CHX chase assay (0, 2, 4, 8 h) examining KDM1A protein stability. (D) Cellular GSH/GSSG ratio. (E) Cellular GPX activity. *n* = 3. (F) Cellular GSH/GSSG ratio. (G) Cellular GPX activity. *n* = 6. *p < 0.05; **p < 0.01; ***p < 0.001.
**Table S1:** The primers used in this study.
**Table S2:** Detailed residue pairs involved in the RAF1‐KDM1A interaction identified by molecular docking.

## Data Availability

The data that support the findings of this study are available from the corresponding author upon reasonable request.
