# Supplementary material for: RAF1 Promotes Gstt1‐Mediated Ferroptosis by Promoting KDM1A Recruitment to Facilitate Pilocarpine‐Induced Acute Seizure‐Associated Neuronal Injury
Source: CNS Neurosci Ther. 2026 Oct 10;32(10):e71177. doi: 10.1002/cns.71177 (PMC13653358; doi:10.1002/cns.71177)
Supplement: Supplementary file 1 [file CNS-32-e71177-s001.docx]

**Supplementary Materials**

**Supplementary Figures**

**Supplementary Figure S1. Cellular localization of RAF1 in the hippocampus of epileptic mice.** (A) RAF1 with the neuronal marker NeuN. Scale bar=25 μm. (B) RAF1 with the astrocytic marker GFAP. Scale bar=25 μm. (C) RAF1 with the microglial marker Iba1. Scale bar=25 μm. (D) RAF1 with the inhibitory neuronal marker GAD67. Scale bar=25 μm. n=3.

**Supplementary Figure S2. Downregulation of RAF1 ameliorates excitotoxic injury in low-Mg^2+^-treated primary hippocampal neurons.** (A) qRT-PCR analysis of RAF1 mRNA expression. (B) Intracellular calcium overload assessed by the Fluo-4 AM calcium fluorescence probe. (C) CCK-8 assay. (D) LDH cytotoxicity assay. (E) Representative images and statistical analysis of Calcein-AM/PI staining. Scale bar=50 μm. (F) 8-OHdG level measurement of DNA damage in cells. n=3. **P*<0.05; ***P*<0.01; ****P*<0.001.

**Supplementary Figure S3. Gstt1 mediates the anti-ferroptotic effect of RAF1 knockdown in glutamate-treated HT22 cells.** (A) qRT-PCR and WB analysis of Gstt1 expression. (B) CCK-8 assay. (C) LDH cytotoxicity assay. (D) The kit was used to detect the content of Fe^2+^ in cells. (E) The expression of ferroptosis-related proteins was detected by WB. (F, H) Cellular GSH/GSSG ratio. (G, I) Cellular GPX activity. n=3. **P*<0.05; ***P*<0.01; ****P*<0.001.

**Supplementary Figure S4. KDM1A mediates RAF1-mediated epigenetic regulation of Gstt1 and functional validation.** (A) WB analysis of KDM1A protein expression. (B) IF staining showing the subcellular localization of KDM1A. Scale bar= μm. (C) CHX chase assay (0, 2, 4, 8 h) examining KDM1A protein stability. (D) Cellular GSH/GSSG ratio. (E) Cellular GPX activity. n=3. (F) Cellular GSH/GSSG ratio. (G) Cellular GPX activity. n=6. **P*<0.05; ***P*<0.01; ****P*<0.001.

**Supplementary Methods**

**Animals**

SPF-grade male C57BL/6 mice, 6 weeks old, were ordered from Hunan SJA Laboratory Animal Co., Ltd. At the start of the experiment, 6 mice were assigned to the Normal group, and 10 mice were assigned to each of the remaining groups, totaling 86 mice. These mice were randomly divided into 8 groups: Normal group, Model group, LV-NC group, LV-sh-RAF1-1/2 group, LV-sh-Gstt1 group, and LV-sh-RAF1+LV-sh-Gstt1 group. Except for the Normal group, the remaining mice were all used to establish a pilocarpine-induced acute seizure-associated neuronal injury (Pilo-ASNI) model. The model construction was consistent with previous studies ^1-4^. Briefly, before inducing epilepsy, scopolamine was injected intraperitoneally into each mouse at a dose of 1 mg/kg (HY-N0296, MCE, USA) to reduce systemic side effects. Twenty minutes after this pretreatment, pilocarpine was administered intraperitoneally at a dose of 300 mg/kg (HY-B0726AR, MCE) until status epilepticus (SE) occurred. Mice in the Normal group were injected with the same volume of saline. The general behavioral activity of the mice was monitored and recorded by a camera, and then analyzed by two researchers who were blinded to the treatment conditions. Seizure onset was defined as the occurrence of stage 4 to 5 seizures according to the Racine scale criteria ^5^. Two hours after seizure onset, SE was terminated by intraperitoneal injection of diazepam (10 mg/kg, 1185008, Sigma, USA) to minimize mortality. Mice were continuously observed for 30 min after diazepam administration to confirm successful termination, during which no sustained generalized seizures were observed. Mice that did not reach stage 4 to 5 seizures within 30 min after scopolamine injection, even after two repeated administrations, were excluded from subsequent studies. In addition, mice that died during seizures (n = 4) or developed severe complications, such as persistent self-injurious behavior, refractory infection, or body weight loss exceeding 20% of initial body weight (n = 2), were excluded. The overall mortality rate was 6/86, approximately 7%. Finally, 6 mice per group were retained for subsequent statistical analysis, with a total of 48 mice included in the study. Seizure severity scoring was again performed during the long-term observation phase following model induction

Two weeks before modeling, lentivirus, including LV-sh-NC, LV-oe-NC, LV-sh-RAF1, and LV-sh-Gstt1 (Genechem, China), was injected into the dorsal dentate gyrus area of the bilateral hippocampus of mice, with 5×10^8^ TU/ML of virus injected into each side, and the injection volume on each side was 3 µl. On the 35th day after SE, the mice were euthanized by cervical dislocation after anesthesia ^6^, and brain tissue and serum samples were collected for later use. All animal handling and experimental protocols were reviewed and approved by the Animal Use and Care Committee‌ of Hainan University (No. HNUAUCC-2025-00144).

**Cell culture and treatment**

Mouse hippocampal neuron cell line HT22 (AW-CNM116, Abiowell, China) was cultured in DMEM medium containing 10% FBS and in a 37°C, 5% CO_2,_ saturated humidity incubator.

Because excessive glutamate release induces neuronal excitotoxicity and oxidative stress, both of which are implicated in epilepsy ^7,8^, HT22 cells were treated with 5 mM glutamate (G6204, Macklin, China) for 24 h to establish an *in vitro* oxidative neuronal injury model. After transfection with sh-NC, oe-NC, sh-RAF1, oe-RAF1, sh-Gstt1, sh-KDM1A, KDM1A-WT and KDM1A-K277A plasmids for 48 h using the transfection reagent LIP2000 (11668019, Invitrogen, USA), the cells were exposed to glutamate. The HT22 cells in the control group were not treated.

Primary hippocampal neuron isolation and Mg^2+^-free seizure-like discharge model *in vitro*: Primary hippocampal neurons were isolated from neonatal (P0-P1) C57 mice. Briefly, brains were rapidly removed into ice-cold D-Hank's solution (pH 7.2, without Ca^2+^/Mg^2+^). Hippocampi were dissected under a microscope, minced into ~1 mm^3^ fragments, and digested with 0.125% trypsin at 37°C for 20 min. Digestion was terminated with plating medium, followed by two washes. Cells were dissociated by gentle pipetting and centrifuged at 1000 rpm for 10 min at 4°C. The pellet was resuspended and filtered through a 200-mesh sieve. Cells were cultured in specialized medium (iCell-n006-002m, iCell, China) at 37°C in a humidified incubator with 5% CO_2_. After transfection with sh-NC or sh-RAF1, cells were incubated for 2 h in Mg^2+^-free buffer (125 mM NaCl, 2.5 mM KCl, 1.25 mM KH_2_PO_4_, 2 mM CaCl_2_, 30 mM glucose, 25 mM HEPES, pH 7.4) to establish a seizure-like discharge model *in vitro* ^9^.

**Immunofluorescence (IF)**

After antigen retrieval and blocking, paraffin sections were incubated with primary antibody RAF1 (1:200, 51140-1-AP, Proteintech, China) or NeuN (1:800, AWA10318, Abiowell) at 4℃ overnight. Subsequently, the sections were incubated with HRP-conjugated secondary antibody, and then labeled with TSA-520 (AWI0688, Abiowell). After DAPI counterstaining, the slides were sealed, and images were collected under a fluorescence microscope.

**Quantitative real-time PCR (qRT-PCR)**

Total RNA was extracted using the TRIzol (15596026, Thermo, USA) method, reverse transcribed into cDNA, and then real-time fluorescence quantitative PCR was performed using the SYBR Green method. β-actin was used as an internal reference, and the relative expression levels of RAF1, Gstt1 and KDM1A were calculated by the 2^−ΔΔCt^ method. Primers are shown in **Supplementary** **Table S1**.

**Western blot (WB)**

Total protein from cells or tissues was extracted using RIPA lysis buffer (P0013B, Beyotime, China), and the protein concentration was determined using the BCA method. Equal amounts of protein samples were subjected to SDS-PAGE electrophoresis and then transferred to a Nitrocellulose (NC) membrane. After blocking with 5% skim milk powder, the specific primary antibody was incubated at 4°C overnight. RAF1 (26863-1-AP, 1:1000, Proteintech), Gstt1 (ab199337, 1:1000, Abcam), GPX4 (AWA11352, 1:1000, Abiowell), SLC7A11 (26864-1-AP, 1:2000, Proteintech), ACSL4 (AWA12612, 1:1000, Abiowell), Ptgs2 (27308-1-AP, 1:3000, Proteintech), KDM1A (20813-1-AP, 1:8000, Proteintech), H3K4me1 (710795, 1:1000, Invitrogen), H3K4me2 (710796, 1:1000, CST), H3K9me2 (49-1007, 1:1000, Proteintech). Then, the membranes were incubated with HRP-conjugated secondary antibody (SA00001-1, 1:5000, anti-mouse, Proteintech or SA00001-2, 1:6000, anti-rabbit, Proteintech) at room temperature for 90 min. Finally, the ECL chemiluminescence (K-12045-D50, Advansta, USA) solution was used for development, and images were collected using the imaging system. β-actin (66009-1-Ig, 1:5000, Proteintech) was used as an internal reference to analyze the relative expression level of the target protein.

**Hematoxylin–Eosin staining (H&E) and Nissl staining**

Paraffin sections were baked, dewaxed and hydrated, and then H&E staining and Nissl staining were performed, respectively. H&E staining was performed with hematoxylin (H8070, Solarbio, China) for nuclear staining and eosin (AWI0029a, Abiowell) for cytoplasmic staining. After gradient ethanol dehydration and xylene transparency, the sections were sealed for observation of tissue morphology. Nissl staining was performed using a Nissl staining kit (AWI0501a, Abiowell). After staining and differentiation, Nissl bodies were purple and sealed with buffered glycerin. All sections were observed under an optical microscope (BA210T, Motic, China), and images were collected.

**Cell Counting Kit-8 (CCK-8)**

Cells were seeded into 24-well plates at a density of 5×10^3^ cells/well, with three replicates per group. After treatment, 300 µL of medium containing CCK-8 reagent (CK04, DOJINDO, Japan) was added to each well and incubated for 4 h. The absorbance at 450 nm was measured with a microplate reader (MB-530, Heales, China), and the average value of the multiple wells was taken for analysis.

**Biochemical detection**

LDH levels were measured using a lactate dehydrogenase cytotoxicity assay kit (C0016, Beyotime, China). The cell culture supernatant was taken, and lactic acid solution, enzyme solution and freshly prepared INT solution were added and incubated at room temperature in the dark for 30 min. The absorbance was measured at 490 nm using a microplate reader. The percentage of cytotoxicity was evaluated by calculating the difference in absorbance between the treatment group and the control group.

The intracellular Fe^2+^ content was determined by the ferrozine colorimetric method (JL-T1255, JONLN, China). After cell lysis and centrifugation, the supernatant was taken and reacted with the color developing reagent at room temperature for 15 min, and the absorbance was measured at a wavelength of 562 nm. The content of Fe^2+^ in the sample was calculated according to the Fe^2+^ standard curve.

Cellular GSH/GSSG ratio was measured using a commercial kit (A061-1-2, Nanjing Jiancheng, China), and GPX activity was determined using a colorimetric assay kit (ab102530, Abcam, UK), following the manufacturers' instructions. GSH/GSSG ratio was calculated based on T-GSH and GSSG contents measured by the DTNB recycling assay at 405 nm. GPX activity was monitored by the decrease in NADPH absorbance at 340 nm and expressed as mU/mg protein.

**Enzyme-linked immunosorbent assay (ELISA)**

The level of 8-OHdG was detected by an ELISA kit (CSB-E10527m, Cusabio, China). After the standard was diluted in series, it was added to the pre-coated plate together with the sample and incubated at 37℃ for 30 min. After washing, the substrate was added for color development and termination, and the absorbance was measured at 450 nm. The sample concentration was calculated according to the standard curve.

**Calcein-AM/PI staining**

Cells in the logarithmic growth phase were seeded in 12-well plates at a density of 10^4^/cm^2^. After treatment, the cells were washed with PBS, and the working solution of live/dead cell staining containing 2 μM Calcein-AM and 8 μM PI (KGAF001, KeyGEN, China) was added and incubated at room temperature in the dark for 30-45 min. The staining solution was removed, and the cells were observed under a fluorescence microscope (AE31E, Motic, China). Calcein-AM labels live cells (green fluorescence), and PI labels dead cells (red fluorescence).

**C11-BODIPY staining**

After treatment, cells were resuspended in PBS containing 2 µM C11-BODIPY 581/591 probe (S0043S, Beyotime) and incubated at 37°C for 20 min in the dark. After washing with PBS, the level of lipid reactive oxygen species (ROS) was detected by flow cytometry.

**RNA sequencing (RNA-seq)**

Transcriptome sequencing and analysis in this study were completed by Shanghai OE Biotech. Co., Ltd. Specifically, the HT22 cell samples treated with glutamate and intervened with sh-RAF1 (with three biological replicates per group) were sequenced using the Illumina NovaSeq 6000 sequencing platform, generating approximately 44–48 million raw reads per sample with a mapping rate of 96.49%–97.42%. cDNA library was constructed using the VAHTS Universal V10 RNA-seq Library Prep Kit. For data analysis, the differentially expressed genes (DEGs) were screened by DESeq2 software (screening threshold: q-value <0.05 and |log_2_FC|>1). Compared with the control group, 710 DEGs (185 upregulated and 525 downregulated) were identified in glutamate-treated HT22 cells. Additionally, in HT22 cells subjected to RAF1 knockdown following glutamate treatment, 109 DEGs (37 upregulated and 72 downregulated) were identified compared with the sh-NC control group. Hierarchical clustering analysis of the obtained DEGs was carried out using the R language to show their expression patterns, and the gene set enrichment analysis (GSEA) was carried out using GSEA software to systematically clarify the regulatory mechanism of RAF1 downregulation in glutamate-induced neuronal injury. The RNA-seq data have been deposited in the Gene Expression Omnibus (GEO) database under accession number PRJNA1512689.

**Co-immunoprecipitation (Co-IP)**

Protein interaction was detected by immunoprecipitation. The cell lysate was incubated with RAF1 antibody (26863-1-AP, 1:1000, Proteintech) at 4°C overnight, and then Protein A/G agarose beads were added and incubated for another 2 h. After elution, WB analysis was performed, and KDM1A (AWA00748, 1:1000, Abiowell) and RAF1 antibodies were used for detection.

**Molecular Docking**

The docking study of protein KDM1A and protein RAF1 was performed using ClusPro software, with docking models ranked by the Docking Score ^10^. Subsequently, the visualization analysis was performed using PYMOL and LigPlus.

**Chromatin Immunoprecipitation followed by quantitative PCR (ChIP-qPCR)**

Cells were cross-linked with formaldehyde and sonicated to obtain chromatin fragments. H3K4me2 (84747-1-RR) or KDM1A (ab129195) antibody was added for immunoprecipitation, and Protein A/G beads were used to capture the complex. After de-crosslinking and DNA purification, qRT-PCR was performed to analyze the enrichment of H3K4me2 or KDM1A in the Gstt1 promoter region. Two independent primer sets targeting the Gstt1 promoter were used for ChIP-qPCR: Set 1: forward: 5’-TCAGCAACCTCCAGACAA-3’, reverse: 5’-AGCACAGATGCCCTACCT-3’; Set 2: forward: 5’-CCAAGGCCATAGAAAGAA-3’, reverse: 5’-CAAGACTGAGTGCCGATT-3’.

**Supplementary Tables**

**Supplementary Table S1. The primers used in this study.**

| **Names** | **Sequences (3'-5')** | **Product length** |
| --- | --- | --- |
| β-actin-F | ACATCCGTAAAGACCTCTATGCC | 223 bp |
| β-actin-R | TACTCCTGCTTGCTGATCCAC |  |
| *Raf1*-F | ATGGCAAGCTCACGGATTCT | 104 bp |
| *Raf1*-R | TGTAAGCTCATTCCATTCCGC |  |
| *Gstt1*-F | TGTCCCGCTCTGTCTGAAAC | 126 bp |
| *Gstt1*-R | GGTGAAGCCACCATCCATCA |  |

-F: forward primer; -R: reverse primer.

**Supplementary Table S2. Detailed residue pairs involved in the RAF1-KDM1A interaction identified by molecular docking.**

| **RAF1 Residue**  **_mouse** | **KDM1A Residue**  **_mouse** | **RAF1 Residue**  **_human** | **KDM1A Residue**  **_human** |
| --- | --- | --- | --- |
| ARG 495 | THR 49 | ASN 617 | GLU 98 |
| LYS 367 | THR 22 | LYS 615 | GLU 98 |
| LYS 309 | GLY 789 | ARG 256 | GLU 103 |
| ARG 401 | GLN 639 | THR 239 | GLU 380 |
| ARG 401 | ARG 592 | THR 234 | GLU 387 |
| GLN 422 | THR 611 | ASN 241 | GLU 373 |
| HIS477 | THR 611 | SER 233 | SER 111 |
| SER 347 | ASN 718 | SER 233 | LYS 114 |
| HIS 369 | ARG 759 | TYR 232 | LYS 114 |
| THR 481 | SER 612 | TYR 232 | ASP 559 |
| ARG 333 | GLU 646 | ARG 223 | ASP 186 |
| ASN 298 | LYS 277 | ARG 231 | ASP 559 |
| PRO 297 | LYS 277 | ARG 231 | HIS 394 |
| ASN 299 | LYS 277 | ARG 231 | ASP 563 |
| ARG 318 | PRO 97 | HIS 230 | ASP 563 |
| ARG 318 | GLU 99 | GLN 229 | PRO 105 |
| HIS 271 | GLN 613 | GLN 229 | GLU 565 |
| LEU270 | GLN 613 | THR 258 | ASN 129 |
| LEU 270 | THR 614 | GLU 219 | LYS 208 |
| SER 291 | ARG 270 | THR 260 | ASN 129 |
| ALA 292 | ILE 276 | SER 259 | SER 131 |
| SER 295 | ILE 276 | SER 259 | GLU 134 |
| ARG 554 | THR 46 | ARG 254 | GLU 138 |
| SER 289 | GLN 581 | HIS 236 | ARG 384 |
| ALA 288 | GLN 581 | SER 227 | ARG 115 |
| ARG 316 | GLU 104 | SER 222 | GLU 175 |
| ARG 316 | ILE 102 | ARG 143 | TYR 135 |
| - | - | LYS 106 | GLU 49 |
| - | - | HIS 103 | GLU 49 |
| - | - | GLU 248 | LYS 149 |
| - | - | THR 631 | THR 88 |

**Supplementary References**

1.	Tong X, Tong Y, Zheng J, et al. TRPM7 contributes to pyroptosis and its involvement in status epilepticus. *Journal of Neuroinflammation.* 2024;21(1).

2.	Zhang K, Yang Z, Yang Z, et al. The m6A reader YTHDC2 promotes the pathophysiology of temporal lobe epilepsy by modulating SLC7A11-dependent glutamate dysregulation in astrocytes. *Theranostics.* 2024;14(14):5551-5570.

3.	Liu L, Wang Y, Wang X, et al. Transient receptor potential vanilloid 4 blockage attenuates pyroptosis in hippocampus of mice following pilocarpine‑induced status epilepticus. *Acta Neuropathologica Communications.* 2025;13(1).

4.	Wang Z, Zhou L, An D, et al. TRPV4-induced inflammatory response is involved in neuronal death in pilocarpine model of temporal lobe epilepsy in mice. *Cell Death & Disease.* 2019;10(6).

5.	Zhong X, Chen L, Wang Y, et al. METTL14/YTHDC1-Mediated m6A Modification in Hippocampus Improves Pentylenetetrazol-Induced Acute Seizures. *Molecular Neurobiology.* 2024;61(12):10979-10991.

6.	Xu B, Zhao X, Luo C, Zhang K, Song C. Inhibiting the NF-κB/DRP1 Axis Affords Neuroprotection after Spinal Cord Injury via Inhibiting Polarization of Pro-Inflammatory Microglia. *Frontiers in Bioscience-Landmark.* 2024;29(8).

7.	Wang Y, Li C, Xiong Z, et al. Up-and-coming anti-epileptic effect of aloesone in Aloe vera: Evidenced by integrating network pharmacological analysis, in vitro, and in vivo models. *Frontiers in Pharmacology.* 2022;13.

8.	Chen S, Zou Q, Guo Q, et al. SPARC Knockdown Reduces Glutamate-Induced HT22 Hippocampal Nerve Cell Damage by Regulating Autophagy. *Frontiers in Neuroscience.* 2021;14.

9.	Mele M, Vieira R, Correia B, et al. Transient incubation of cultured hippocampal neurons in the absence of magnesium induces rhythmic and synchronized epileptiform-like activity. *Scientific Reports.* 2021;11(1).

10.	Ehsasatvatan M, Baghban Kohnehrouz B. Designing and computational analyzing of chimeric long-lasting GLP-1 receptor agonists for type 2 diabetes. *Scientific Reports.* 2023;13(1).
